# Addressing Critical Fungal Pathogens Under a One Health Perspective: Key Insights from the Portuguese Association of Medical Mycology

**DOI:** 10.1007/s11046-025-00981-3

**Published:** 2025-08-16

**Authors:** R. Sabino, F. Antunes, R. Araujo, A. R. Bezerra, J. Brandão, C. Carneiro, A. Carvalho, D. Carvalho, I. C. Conceição, F. Cota Medeiros, C. Cruz, E. Duarte, S. Holum, O. Matos, F. Maltez, A. Mendonça, G. Moura, A. Pereira, C. Fortuna Rodrigues, P. Teixeira, S. R. Valdoleiros, C. Veríssimo, C. Viegas, The Portuguese Association of Medical Mycology (ASPOMM)

**Affiliations:** 1 Faculdade de Farmácia, Universidade de Lisboa, Lisbon, Portugal; 2 Instituto de Saúde Ambiental, Faculdade de Medicina, Universidade de Lisboa, Lisbon, Portugal; 3 Laboratório Associado TERRA, Faculdade de Medicina, Universidade de Lisboa, Lisbon, Portugal; 4 i3S—Instituto de Investigação e Inovação em Saúde, Universidade do Porto, Porto, Portugal; 5 INEB—Instituto de Engenharia Biomédica, Universidade do Porto, Porto, Portugal; 6 Institute of Biomedicine (iBiMED) and Department of Medical Sciences, University of Aveiro, Aveiro, Portugal; 7 Department of Environmental Health, National Institute of Health Doutor Ricardo Jorge, 1649-016 Lisbon, Portugal; 8 cE3c – Centre for Ecology, Evolution and Environmental Changes, Faculdade de Ciências da Universidade de Lisboa, Campo Grande 016, 1749-016 Lisbon, Portugal; 9 Escola Superior de Tecnologia de Setúbal, Instituto Politécnico de Setúbal, Campus Do IPS, 2910-761 Setúbal, Portugal; 10 Life and Health Sciences Research Institute (ICVS), School of Medicine, University of Minho, Braga, Portugal; 11 ICVS/3B’s – PT Government Associate Laboratory, Braga/Guimarães, Portugal; 12 Serviço de Patologia Clínica, Unidade Local de Saúde Santa Maria, Lisbon, Portugal; 13 Laboratório de Microbiologia Clínica, Hospital do SAMS, Lisbon, Portugal; 14 Instituto de Microbiologia, Faculdade de Medicina, Universidade de Lisboa, Lisbon, Portugal; 15 Serviço de Doenças Infecciosas, ULS Santa Maria, Lisbon, Portugal; 16 Serviço de Doenças Infeciosas do Hospital de Curry Cabral –ULS São José, Lisbon, Portugal; 17 MED - Mediterranean Institute for Agriculture, Environment and Development & Department of Veterinary Medicine, ECT, Universidade de Évora, Apartado 94, 7002-554 Évora, Portugal; 18 Global Health and Tropical Medicine, GHTM, Associate Laboratory in Translation and Innovation Towards Global Health, LA-REAL, Instituto de Higiene E Medicina Tropical, IHMT, Universidade NOVA de Lisboa, Lisbon, Portugal; 19 NOVA MEDICAL SCHOOL, Lisbon, Portugal; 20 CBMA – Center of Molecular and Environmental Biology, Department of Biology, University of Minho, Braga, Portugal; 21 Clínica Universitária de Doenças Infecciosas, Faculdade de Medicina, Universidade de Lisboa. Avenida Professor Egas Moniz, 1649-028 Lisbon, Portugal; 22 Associate Laboratory i4HB, Institute for Health and Bioeconomy, University Institute of Health Sciences-CESPU, Gandra, Portugal; 23 UCIBIO - Applied Molecular Biosciences Unit, Translational Toxicology Research Laboratory, University Institute of Health Sciences (1H-TOXRUN, IUCS-CESPU), 4585-116 Gandra, Portugal; 24 ALiCE—Associate Laboratory in Chemical Engineering and LEPABE—Laboratory for Process Engineering, Environment, Biotechnology and Energy, Faculty of Engineering, University of Porto, Rua Dr. Roberto Frias, 4200-465 Porto, Portugal; 25 Lisbon Municipality, Municipal Directorate for Environment, Green Infrastructure, Climate and Energy, Bromatology and Water Laboratory, Avenida Cidade Do Porto S/N, 1700-111 Lisbon, Portugal; 26 Infectious Diseases Department, Centro Hospitalar Universitário de São João, Porto, Portugal; 27 Faculty of Medicine, University of Porto, Porto, Portugal; 28 Reference Unit for Parasitic and Fungal Infections, Department of Infectious Diseases, National Institute of Health Doutor Ricardo Jorge (INSA), Lisbon, Portugal; 29 H&TRC—Health & Technology Research Center, ESTeSL—Escola Superior de Tecnologia e Saúde, Instituto Politécnico de Lisboa, 1990-096 Lisbon, Portugal; 30 Public Health Research Centre, Comprehensive Health Research Center, CHRC, REAL, CCAL, NOVA National School of Public Health, NOVA University Lisbon, 1099-085 Lisbon, Portugal

**Keywords:** *Aspergillus*, *Candida*, *Cryptococcus*, Portugal, Medical Mycology, One Health

## Abstract

Fungal infections have emerged as a significant public health concern, especially with the increasing incidence of severe mycoses caused by pathogens such as *Aspergillus fumigatus*, *Candida auris*, *Candida albicans*, and *Cryptococcus neoformans*. These fungi, listed as critical priorities by the World Health Organization, pose a heightened risk due to rising antifungal resistance and their severe impact on immunocompromised individuals. This article, coordinated by the Portuguese Association of Medical Mycology, highlights the importance of adopting a One Health perspective to address fungal threats comprehensively. Drawing on interdisciplinary collaboration, the association aims to foster greater awareness, improve diagnostic capabilities, and stimulate research and public health policies in Portugal but also at global level. The paper outlines key strategies for surveillance, prevention, and innovation in fungal diagnostics and therapeutics. Moreover, it emphasizes the urgent need for national coordination and international cooperation in managing fungal infections, advocating for integrative approaches that link human, animal, and environmental health. By presenting a consolidated overview of current challenges and future priorities, this work seeks to enhance preparedness and response mechanisms in the face of escalating fungal threats.

## Introduction

In 2022, the World Health Organization (WHO) established a priority list of fungal pathogens based on the urgency for knowledge gathering and intervention to protect human health [1]. As described in its presentation, *“The WHO fungal priority pathogens list (WHO FPPL) is the first global effort to systematically prioritize fungal pathogens, considering their unmet research and development (R&D) needs and perceived public health importance”*. *Aspergillus fumigatus, Candida auris, Candida albicans* and *Cryptococcus neoformans* were assigned the highest priority level: critical. These microorganisms have been garnering increasing attention due to their ability to cause severe infections, with high mortality rates, particularly in immunocompromised people, and their growing resistance to antifungal drugs, which complicates treatment and management strategies.

Fungal infections represent a significant public health challenge around the world. Countries are thus encouraged to improve their mycological diagnostic capacity, to promptly and efficiently manage fungal infections and to perform surveillance towards a deeper knowledge of their epidemiology [1]. The “One Health" framework associated with these four critical pathogens emphasizes the interconnectedness of human, animal, and environmental health. Aiming to meet the WHO recommendations afore mentioned, the Portuguese Association of Medical Mycology (ASPOMM), gathering experts from different clinical and scientific areas, performed a national study on current actions and studies, focused on the critical species. The study highlights the unmet needs to promote local knowledge on fungal infections caused by these specific agents, their epidemiological distribution and antifungal resistance patterns. Additionally, ASPOMM aimed to raise awareness to other emergent issues regarding severe fungal infections, such as the increase of their incidence and major factors that trigger this emergence.

This expanded article delves deeply into the prevalence, resistance patterns, diagnostic capabilities, therapeutic challenges, and environmental niches of these critical fungal pathogens, emphasizing the need for a multidisciplinary approach. With this publication, ASPOMM aims to address public health needs by informing and empowering policymakers in the design and implementation of future measures to combat invasive fungal diseases and antifungal resistance.

## Overview of Critical Fungal Pathogens in Portugal

Fungal pathogens of critical concern in Portugal are diverse in their biology, mechanisms of pathogenicity, and clinical implications. In the following sections, the four critical pathogenic fungi will be discussed under the Portuguese reality.

The most frequent etiological agent of infection by filamentous fungi is ***Aspergillus fumigatus***, a ubiquitous mold originating aspergillosis. This fungal disease often results from an allergic response to *Aspergillus* spp. or, less frequently, as the result of tissue and vascular invasion by this fungus. As consequence, it leads to multiorgan dysfunction (mainly the airways and lungs, but also extrapulmonary dissemination) [2, 3]. In invasive extrapulmonary aspergillosis, *Aspergillus* spp. can disseminate beyond the respiratory tract to multiple organs, especially in severely immunocompromised patients, with a very poor prognosis and high mortality rate [2, 4]. Examples of these are the central nervous system (CNS) aspergillosis and cutaneous aspergillosis, endophthalmitis, and endocarditis. *Aspergillus* spp. is the second cause of fungal endocarditis after *Candida* spp. and primarily occurs in patients with prosthetic heart valves (due to contamination during surgery). This potentially pathogenic fungus is particularly dangerous for immunosuppressed individuals, such as those undergoing chemotherapy or organ transplantation [5].

In 2017, a paper on severe fungal infections in Portugal [6] published for the first time the estimated burden of fungal diseases using a model that considered specific populations at risk of *Aspergillus* infection. An incidence rate of 2.3/100,000 was determined. In 2022, the Portuguese National Institute of Health Dr. Ricardo Jorge (NIH) ran a laboratory survey to characterize the etiology of Invasive and Subcutaneous Fungal Infections—the ‘IFIs’ network [7]. Fourteen Portuguese laboratories were enrolled, and 76 reports were validated. *Aspergillus* was the most frequent fungal genus detected (n = 15; 19.7%), being more frequently identified in the proven and probable invasive fungal infections (IFI) cases. *Fumigati* was the section most frequently found (n = 11), representing 20.8% of the etiological agents, causing proven and probable invasive fungal infections (n = 53). In this section, the species *A. fumigatus* sensu stricto was the most frequently detected, but a *A. felis/parafelis* isolate was also identified in one case. Additionally, a laboratory-based national surveillance program for *Aspergillus* established in Portugal, since 2012, and coordinated by the National Reference Laboratory for Parasitic and Fungal Infections of the Portuguese NIH, collected data regarding molecular epidemiology of *Aspergillus* and antifungal susceptibility profile of *Aspergillus* section *Fumigati* [8]. From the 256 isolates collected during the study period (2017–2018), 156 were from human sources and 99 from different environmental sources and *A. fumigatus* sensu stricto was the most frequent species in both human and environmental sources.

***Candida albicans*** is the main agent responsible for mucosal disease, with an estimated global burden of ~ 134,000,000 recurrent vulvovaginal candidiasis [9], and more than one million annual incidence of oral or esophageal candidiasis worldwide associated with human immunodeficiency virus (HIV) infection [10]. *Candida albicans* is also the most widespread pathogen identified in the majority of candidemia cases worldwide [11] and it is the most commonly isolated fungal pathogen in Portugal, associated with a spectrum of diseases ranging from superficial mucosal infections to invasive bloodstream infections (candidemia). Reports from Portuguese hospitals indicate a higher prevalence of *C. albicans* infections in the northern and central regions, with significant burdens observed in oncology and neonatal units. In a multicentric observational study [11] performed during 24 months (2010–2011), and enrolling all candidemia cases detected in 10 hospitals from northern (4), central (2), and southern (4) regions of Portugal, the mean incidence of fungemia was 0.88 per 1,000 admissions, ranging from 0.15 to 2.4, depending on the hospital. The mean incidence of nosocomial fungemia was 0.74 per 1,000 admissions, ranging from 0.14 to 2.1. Of the 240 episodes of fungemia, *C. albicans* (40%) was the most prevalent, followed by *C. parapsilosis* (23%) and *C. glabrata* (13%). In Portugal, candidemia affects 2.19 per 100,000 patients, totaling approximately 231 cases annually [6]. A multicenter survey reported an incidence of 0.88 per 1,000 hospital admissions, with *C. albicans* as the predominant species, followed by *C. glabrata* and *C. parapsilosis* [12, 13]. In a tertiary care hospital study, 117 episodes of candidemia were documented over two years with a 30-day mortality rate of 31.6% [14]. Interestingly, in a single center study published in 2010 [13], on oncological patients specifically, *C. albicans* was more frequently associated with solid tumors of the gastrointestinal and genitourinary tracts and breast (*p* = 0.005), while non-*albicans* species were most frequently recovered from hematological patients (*p* = 0.007). Also, a retrospective study concerning a two-year period, that included all patients from a Portuguese tertiary care hospital, observed a 51.3% prevalence of *C. albicans* isolates in 117 candidemia episodes [14]. The main risk factors for the isolation of *C. albicans* were previous antibacterial therapy and the presence of central venous catheter. Similarly to what Sabino et al. (2010) observed [13], *C. albicans* was more prevalent in oncological patients with solid tumors (53%), compared with hematological malignancies (28%).

According to the paper published in 2017 on serious fungal infections in Portugal [6] and following a statistical model that considered specific populations at risk of *Candida* infection, the following rates were calculated: candidemia 2.2/100,000; *Candida* peritonitis 0.37/100,000; oral candidiasis 20/100,000; oesophageal candidiasis 17/100,000; and recurrent *Candida* vaginitis (> 4 × /year) 2853/100,000.

In 2019, another study [15] examined the frequency of isolation of *Candida* spp. in more than 1000 isolates collected from sterile and non-sterile samples in five major hospital centers and in a set of collection stations of the Lisbon area. Of the 1030 isolates of the genus *Candida*, 42 came from sterile products and 989 came from non-sterile products. *C. albicans* was the most frequent isolated species, with a prevalence of around 74% in both types of samples.

In 2023, in an 11-years study [16] on *Candida* vulvovaginal infection performed in one hospital, the overall prevalence of *Candida* spp. in vaginal swabs was of 19.4% (n = 148). Additionally, there was a prevalence of vulvovaginal candidiasis of 20.5% (n = 116), and of asymptomatic colonization of 16.1% (n = 32). The most frequently isolated species was *C. albicans* in both symptomatic and symptom-free women, with a prevalence of 90.5% and 68.8%, respectively. Moreover, in a three-year retrospective study (2014–2016), including 12 Portuguese hospitals that studied the epidemiology of superficial fungal infections [17], yeasts corresponded to 13% of the total isolated agents in all samples, with *C. albicans* being the most prevalent (38.5%).

Also considered critical is *C. auris (*new name ***Candidozyma auris)***, which poses several challenges, since it is difficult to eradicate from hospitals, being resistant to disinfection measures, and can cause life threatening infections. In hospitals, it is mainly found on the skin of staff and surfaces. So far, six different genetic clades have been identified (I to VI), each exhibiting a specific antifungal resistance pattern. Between 2020 and 2022 [18], a prospective multicenter study was conducted in two Intensive Care Units (ICUs) of tertiary care hospitals in the Lisbon region, aiming to monitor the local epidemiology of *Candida* species and identify the prevalence of *C. auris*. A total of 988 samples from 675 patients were analyzed. Although various *Candida* species were identified, *C. auris* was not detected, which is interesting, since Spain, our neighboring country, is endemic for this species. This is backed up by the very faint and occasional detection in community and hospital wastewater monitoring of Lisbon (ongoing research). Kohlenberg et al. [19] mentioned that one Portuguese case was reported to the European Centre for Disease Prevention and Control in 2022, but no further information was available in the publication. In 2023, the first case of *C. auris* isolation from a patient in Portugal was reported [20]. It was isolated from a bronchoalveolar lavage sample of a patient transferred from Angola to an ICU in Lisbon, Portugal for a liver transplant after a SARS-CoV-2 infection. Genomic analysis revealed that the isolate belonged to Clade III, genetically related to African isolates. Following this case, the first reports of *C. auris* at the largest hospital in northern Portugal were detected associated to both candidemia and colonization. All strains were classified as Clade I (South Asian). (https://www.ncbi.nlm.nih.gov/bioproject/1140147). This low frequency of isolation of *C. auris* may not reflect the real situation of the country since the isolation of this agent is not of mandatory declaration to the Health Authorities. Nevertheless, these cases highlight the need for continuous surveillance in hospital settings for early detection and implementation of efficient control measures.

***Cryptococcus neoformans*** is a significant cause of cryptococcal meningitis, primarily affecting individuals with HIV / acquired immunodeficiency syndrome (AIDS) or those who have undergone organ transplantation. Clinically, cases of cryptococcal meningitis in Portugal are often severe, with high mortality rates attributed to the pathogen’s virulence factors, including melanin production and the formation of a polysaccharide capsule, which enhance its ability to evade the host immune response.

A Portuguese study [21] describes the genotypic characterization of 185 *Cryptococcus* spp. strains from cryptococcosis cases in two hospitals in the Lisboa and the Tagus Valley region in the period 1991–2007. The study identified the presence of the following genotypes: VNI (43.0%), VNII (12.0%), VNIII (33.3%), VGNIV (10.7%), and only one circulating isolate of VGII. In a study that assessed patients from a tertiary hospital in the Lisboa and the Tagus Valley region with diagnosis of cryptococcosis (based on the laboratory isolation of *Cryptococcus* spp.) between the years 2007–2022, 221 isolations of *Cryptococcus* spp. were detected. Excluding duplicates, 119 isolations were identified, corresponding to 71 patients, with a decreasing trend in isolations over time [22]. This period does however incorporate the SARS-CoV-2 pandemic with societal quarantine periods and greater care in avoiding transmission of infections to hospitalized patients. In this case series, an overall incidence of 0.042 per 100,000 inhabitants was obtained. The prevalence of *C. neoformans* was 94.3% (67/71). The remaining identified species account for 5.7% of the isolates, with *C. gattii* found in two patients with immunosuppression associated with HIV infection—one diagnosed with pulmonary cryptococcosis (positive bronchoalveolar lavage) and another with meningocerebral cryptococcosis (positive cerebrospinal fluid) and reported cerebral cryptococcomas. Additionally, *C. deneoformans* was identified in one immunosuppressed patient (allogeneic hematopoietic stem cell transplantation with chronic graft-versus-host disease) with disseminated cryptococcosis presenting with cutaneous manifestations, without CNS involvement [23]. The overall mortality rate was 46.5% (33/71).

## Diagnostic Capabilities and Laboratory Challenges in Portugal

Accurate and timely diagnosis is fundamental to managing fungal infections effectively, yet significant challenges remain in Portugal.

Portugal has a National Reference Laboratory for Parasitic and Fungal infections at the NIH has a solid expertise in clinical and environmental mycology. It provides reference services in the identification of filamentous fungi and yeast strains and determining their susceptibility pattern to antifungals. It works to improve diagnosis of superficial and systemic fungal infections, through conventional, molecular and serological methods. It also possesses an extensive culture collection of environmental and clinical isolates and is currently accredited by the Portuguese Institute for Accreditation (IPAC). The Reference Laboratory has also a long history of advanced training of young researchers (master and PhD students) as well as specialized training to clinical pathologists, infectious diseases and environmental health physicians. Besides the National Reference Laboratory for Fungal infections at the NIH, several tertiary care hospitals and centralized laboratories can perform a diagnosis of fungal infections with expertise. In order to perceive this reality, a multicentric study on the Portuguese clinical mycology capacity was promoted by the International Society of Human and Animal Mycology and the European Confederation for Medical Mycology [24]. This study, published in 2024, enrolled a total of 16 Portuguese institutions that self-assessed their capability to manage invasive fungal infections in Portugal. Laboratories employ a range of techniques to identify fungal pathogens, including culture-based methods, molecular diagnostics, and biomarker detection. Culture-based methods are widely used. For species identification, automated identification through VITEK 2® and other commercial tests was performed by the 16 laboratories (100%), followed by classical biochemical tests (n = 11, 68.8%), matrix-assisted laser desorption ionization time of-flight mass spectrometry (MALDI-TOF MS) (n = 10, 62.5%), deoxyribonucleic acid (DNA) sequencing (n = 6, 37.5%).

Aiming to raise awareness of the risk of misidentification associated to *C. auris*, the Portuguese External Quality Assessment Program (in collaboration with the National Reference Laboratory for Parasitic and Fungal Infections of the Portuguese NIH), organized a pilot study in 2020 to evaluate the ability of Portuguese clinical microbiology laboratories to correctly identify *C. auris*. Seventeen laboratories participated in this study, eight hospital laboratories and nine ambulatory laboratories. *C. auris* was correctly identified by 88% (15/17) of the participating laboratories. One laboratory was not able to identify this species, and another identified it as a different *Candida* species. All the participants from hospital laboratories reported the correct species identification. After this first study, the scheme is distributed once a year to the institutions that participate. Specifically for presumptive detection of *C. auris*, some laboratories incubate a plate at 40ºC and use chromogenic media. For species confirmation, MALDI-TOF MS and sequencing the region D1-D2 of the rDNA are done in some hospitals and in the reference laboratory. In cases of possible *C. auris* detection, the isolate is sent to the Portuguese NIH to perform the gold standard identification by DNA sequencing.

Biomarker assays, including *Cryptococcus* antigen detection, galactomannan and beta-D-glucan tests, are standard tools in Portugal for diagnosing cryptococosis (in 75% of the inquired laboratories), invasive aspergillosis (in 62.5%) and candidemia (in 43.8%), respectively. Molecular diagnosis was not performed in all laboratories. There are notable disparities in diagnostic capabilities across Portugal. Tertiary care hospitals in cities like Lisbon and Oporto are equipped with advanced molecular tools and trained personnel, enabling early detection and effective intervention. In contrast, smaller hospitals and rural healthcare facilities often lack access to these resources, leading to delays in diagnosis and suboptimal patient outcomes. Addressing these gaps requires significant investment in diagnostic infrastructure, the expansion of training programs for laboratory staff, and the development of integrated national surveillance systems to monitor fungal infections comprehensively.

## Antifungal Resistance and its Implications in Portugal

Antifungal resistance poses a significant challenge to managing fungal infections, with resistance mechanisms varying across species. Portugal is one of the countries without a national surveillance system for this purpose, and most studies on antifungal susceptibility are not recent and used small samples, making it impossible to draw conclusions about recent trends of antifungal resistance.

For the study of azole-resistance patterns in *A. fumigatus* in Portugal, the entire collection of isolates identified as belonging to *Fumigati*  section (2012–2019) of the National Reference Laboratory for Parasitic and Fungal Infections at the NIH in Lisbon was analyzed [8], which included both clinical and environmental isolates from different regions of the country. A total of 337 isolates belonging to the *Fumigati* section were analyzed and among these, the frequency of cryptic species was 5.3% (18/337). Regarding only the *Fumigati* cryptic species, its overall resistance to azoles was 33.3% (all resistant isolates were from clinical human sources). The overall prevalence of resistance to azoles in *A. fumigatus* sensu stricto was 3.0% (four clinical and five environmental isolates). The TR34/L98H was the most frequently detected mutation (in 1.4% of the isolates), found in three environmental and one clinical isolate.

Regarding *C. albicans* antifungal resistance, one of the first epidemiological surveys conducted in Portugal took place in one tertiary hospital Northern Portugal, during a 12-month period in 2004 [25]. Forty-one *C. albicans* isolates were retrieved and high levels of azole resistance were reported, specifically 10%, 20%, and 27% for voriconazole, fluconazole, and itraconazole, respectively. The only echinocandin tested was caspofungin with 5% of isolates presenting a non-susceptible profile. All isolates were susceptible to the polyene tested – amphotericin B.

In another study, conducted between 2002 and 2007 in an oncology hospital in Lisbon, 58 strains of *C. albicans* were isolated from blood cultures of cancer patients. No cases of resistance were reported for fluconazole, voriconazole or itraconazole, while caspofungin had a resistance rate of 7.3% [13].

Between 2011 and 2012, 10 hospitals across Portugal provided 97 *C. albicans* isolates collected from blood cultures of patients with fungemia. Azole resistance was 2%, 4% and 11% for fluconazole, voriconazole, and posaconazole, respectively. Resistance to echinocandins ranged from 8% for anidulafungin to 15% for micafungin. All isolates were susceptible to amphotericin B. Resistance to drugs within the same class was observed, with eight isolates of *C. albicans* showing resistance to the three azoles [12].

In a study aiming to assess adhesion, biofilm formation, cell surface hydrophobicity and antifungal susceptibility of 184 *Candida* clinical isolates, 49 were identified as *C. albicans*. Azole resistance was 12%, echinocandin 6% and no resistance to the polyene was detected. Also, no association was found between antifungal resistance and higher adhesion profile or biofilm formation [26].

The most recent multicentric study was conducted between 2020 and 2022 and retrieved 185 *C. albicans* isolates from axillar/inguinal swabs of ICU patients. Azole resistance rates among these isolates was 0.8% for fluconazole and 1.6% for voriconazole, with two isolates showing resistance to both azoles tested. Resistance to anidulafungin was higher (3.8%) while no resistance to amphotericin B was detected [27].

The previously mentioned *C. auris* Portuguese isolates belonging to Clade I had high minimum inhibitory concentrations (MIC) values for fluconazole and amphotericin B and harbored the *erg11* (Y132F) and *cdr11* (E709D) mutations, along with a newly identified *fks1* (I1465L) mutation. The isolate belonging to Clade III was resistant only to azoles and the mutations C125A and F126L in *erg11* gene were detected, both potentially associated with azole resistance [20].

Although these studies provide valuable data on the species distribution and antifungal susceptibility, it is important to highlight the variable geographical location of the centers involved, the extreme variability between studies and their relatively small sample size. Differences in methodology and interpretation criteria are influenced by the constant evolution in the field of resistance identification and consequent changes in the breakpoints defined by both CLSI and EUCAST guidelines [28]. This reinforces the importance of standardizing surveillance system reports, including disease definition, susceptibility patterns and the need of well characterized biobanks to retrospective assessment of isolates. These efforts would strengthen the link between surveillance efforts and antifungal resistance monitoring. Enhancing surveillance data on fungal infections is crucial to accurately determining disease burden, prioritizing research and development, and guiding antifungal stewardship strategies.

## Environmental and Veterinary Aspects of these Fungal Pathogens in Portugal

Collaborative efforts between clinical and environmental researchers are essential to trace the origins of resistance and develop targeted interventions. Additionally, the environmental and animal health dimensions of fungal pathogens are integral to the One Health approach.

*Aspergillus* spp. are ubiquitous [29], widely dispersed in the environment, namely in freshwater and marine habitats, soils, air or biosolids, being able to adapt to a wide range of environmental conditions and to use a broad diversity of organic substrates [30].

When dealing with an environmental fungus capable of infecting both humans and animals, and with a rising problem of resistance of environmental origin, it is also important to better understand how isolates from these sources relate to each other. The understanding of the epidemiology of this fungus allows a better understanding of the interactions of the fungus in the environment and the human body. In a study performed by Morais et al. [31], microsatellite genotyping was performed to 100 viable isolates from different sources (environmental and clinical-human and animal). Although the high genetic diversity of the isolates, some of them, collected from different sources appeared to be closely related, with exactly the same multilocus genotypes. This observation highlights the importance of *A. fumigatus* in the One Health context, since the same multilocus genotype can be shared among environmental and clinical (human and animal) isolates.

Thus, the knowledge of the epidemiology of environmental *Aspergillus* in each hospital may allow the establishment of preventive or corrective measures to decrease nosocomial fungal infections. In a study performed by Sabino et al. [32], in a tertiary hospital during one-year period, four seasonal samplings, of air and hard surfaces, were performed. A total of 101 air samples and 99 surface samples were collected from the Hematology, Oncology, and ICU wards. *Aspergillus* was the most frequently recovered fungal genus (19.7%) in all the three units of immunocompromised patients, with section *Fumigati* representing 8.8% to the total *Aspergillus* isolates.

In a study conducted in 18 medical units from a tertiary hospital of the northern region of Portugal, between 2004 and 2005, *Penicillium, Aspergillus, Scedosporium, Alternaria, Cladosporium, Rhizopus* and *Mucor* were the most frequently isolated airborne molds [33]. An additional study conducted during renovation works at the Hematology ward of the same hospital showed that the levels of *A. fumigatus* might increase during constructions. The fungal levels diminished following the installation of high efficiency particulate air (HEPA) filters within the wards [34]. In fact, the study showed a gradual improvement of the air quality in the new rooms with HEPA filters since the first week, decreasing the total values to less than seven CFU⁄m^3^ after the second week confirming a reduction of more than 95% for total fungi. *A. fumigatus* could not be detected after the second week.

Molds can also be waterborne and appear in biofilms [35], but a study conducted in a tertiary hospital in the North, showed low fungal concentration in the hospital tap water and *A. fumigatus* was detected in less than 6% of the total samples [33]. Characterization of wastewater effluents into wastewater treatment plants (WWTPs) enables processes to be managed with improved efficiency, contributing to environmentally safe treated wastewater, either for final disposal or reuse, not excluding the worrying occupational exposure of WWTP workers (ongoing project). Further studies on *A. fumigatus* prevalence in wastewater are urgent, particularly in settings of interest such as hospitals.

A total of 250 environmental *A. fumigatus* isolates were analyzed for genetic diversity using multiple microsatellite typing [36]. High genotypic diversity (average of 0.95) was usually found in indoor environments from hospital wards. The diversity values did not change within the wards regardless of the use of HEPA filters, other filtration systems (e.g., fine filters) or air-conditioners. The results suggested that very few genotypes could persist indoors after a few months of repetitive sampling but *A. fumigatus* population can easily disperse inside buildings, even when multiple physical barriers and filters are installed. In addition, microvariation events are frequent in indoor environments, showing that these mold populations may evolve over time [36].

Besides clinical settings, other Portuguese indoor environments had been assessed in what concerns fungal contamination and considered as hot spots for *Aspergillus* section *Fumigati* exposure. Apart from waste management industry, where this section was considered as a sentinel for harmful fungal exposure [37], as in other countries such as Canada [38] and Norway [39], also in fitness centers, groceries and “Do-It-Yourself” stores [40] ubiquity was found. These reports point out, not only the need to assess fungal contamination in a broad diversity of settings, but also to employ a wide array of sampling methods (besides air sampling) and analyses, as was the case in all these studies. In fact, it was in protection respiratory devices used by waste sorting workers where, for the first time in Portugal, environmental isolates bearing the TR34/L98H mutation were reported This clearly corroborates the relevance of environmental surveillance on the emergence of azole-resistant *A. fumigatus* sensu stricto strains in occupational environments besides clinical ones, to ensure proper policies risk management measures that may have a positive impact on occupational and public health [41].

In fact, environmental studies conducted in Portugal have highlighted the widespread presence of azole-resistant *A. fumigatus* strains. In a study performed in 2014 [42], antifungal susceptibility testing of environmental and clinical isolates of *A. fumigatus* was performed after exposing *A. fumigatus* to prochloraz (PCZ), an agricultural antifungal. This exposure induced morphological changes in *A. fumigatus*, with increasing MIC values to PCZ along the time as well as the development of cross-resistance with posaconazole, itraconazole and voriconazole. The data obtained in this study correlated the emergence of azole resistances in clinical isolates with the application of agricultural azole fungicides.

In the study performed by Pinto et al. [43] eight azole-resistant *A. fumigatus* sensu stricto isolates were studied. Mutations in the *cyp51A* gene were found in six of them, more precisely: the TR34/L98H and TR46/Y121F/T289A mutations. In all cases, most mutations that confer azole resistance were associated with environmental exposure and that reinforces the importance of *A. fumigatus* detection in specific environments.

Concerning animal health, diagnostics of fungal infections in domestic and wild animals is performed by the mycology laboratory of the Portuguese National Reference Laboratory for animal diseases, laboratories in academia or private diagnostic laboratories. For superficial mycosis such as dermatophytosis, most veterinary practices also perform routine diagnosis (Wood lamp observation, trichograms and culture). Classical/ morphological identification is performed as well as PCR to confirm the identification of filamentous fungi or to identify yeasts. Moreover, for deep mycosis, every histopathology veterinary laboratory performs specific PAS and/or silver staining. Nevertheless, none are equipped to perform antifungal susceptibility testing (AST), which is still infrequently requested by veterinary clinicians, being performed only for research purposes. This gap may lead to a lack of knowledge of the antifungal susceptibility profiles associated with fungal isolates collected from veterinary sources. In Portugal, the presence of *Aspergillus* and other fungi in animal facilities has been assessed as a frequent source of occupational hazards and mycotoxin exposure and contamination of animal feed [32, 44–47] and the facilities promoting exposure of workers, animals and consumers [48, 49].

*Candida* species are also ubiquitous fungi found in various environmental settings in Portugal, including soil, air, water, and indoor environments. Their presence has been documented in healthcare facilities, beach sands, and residential areas. Research assessing the mycological quality of sand beaches in the Portuguese coastal area identified *Candida* species as indicators of environmental quality [50]. It was also found that environmental isolates of the *C. parapsilosis* complex from hospital settings exhibited higher virulence compared to clinical isolates. This underscores the potential risk posed by these environmental strains to fuel nosocomial infections, especially in immunocompromised patients [51].

Epidemiological studies have highlighted the prevalence of *Candida* infections in veterinary settings, where the warm and humid climate may contribute to the fungus’s proliferation [52]. Moreover, the increasing use of antifungal drugs in animal husbandry has led to concerns about the emergence of drug-resistant *Candida* strains, posing a significant challenge to veterinary practitioners. A recent study conducted in Portugal revealed the presence of *C. auris* in household pets, particularly dogs, highlighting the potential for zoonotic transmission and the importance of addressing this issue at the societal level [53]. Also, it has been emphasized the need for improved awareness and education among pet owners regarding the risks associated with antimicrobial resistance and zoonotic diseases [53]. The emergence of *C. auris* in animal populations is not limited to Portugal, as similar observations have been made in other parts of the world [54].

In Portugal, urban environments with dense pigeon populations serve as major reservoirs for *C. neoformans*. Environmental studies have documented contamination with this pathogen in cities like Lisbon and Oporto [55].

Veterinary cases of fungal infections, including cryptococcosis, highlight the zoonotic potential of certain pathogens. Cryptococcosis, especially in cats, is almost exclusively associated with immunosuppression, namely in cats with feline immunodeficiency virus, as previously mentioned in humans and HIV. Morera et al. describes the detection of two cases of cryptococcosis in ferrets in the Iberian Peninsula and Balearic Islands and suggests the presence of a diversity of pathogenic *Cryptococcus* species in the Mediterranean environment. One of the cases, detected in Portugal, is considered particularly relevant since it describes the first local animal infection caused by an isolate belonging to the hypervirulent *C. gattii* AFLP6/VGII genotype. These results remark the important role that this species can play to prevent human cases acting as sentinels of the presence of these yeasts in the environment [56]. While data on fungal infections in livestock and companion animals in Portugal are limited, the findings underscore the need for integrated surveillance systems that encompass both human and animal health. Such systems are crucial for understanding the dynamics of fungal pathogen transmission and developing effective control strategies.

Climate change has a significant role in fungal development, proliferation and distribution, pathogenicity, and the antifungal susceptibility pattern. Climate change effects on fungi lead to new environmental pressures that result in the emergence of novel pathogens in plants, animals, and humans. This can lead to new fungal diseases, which may compromise production yields, public health, and wildlife biodiversity [57, 58]. The International Panel on Climate Change [59] and almost the entire scientific community agreed to consider the Mediterranean Area as one of the most vulnerable regions in the world to the impacts of global warming. In this scenario, heat waves, defined by the World Meteorological Organization as five or more consecutive days of heat reaching a daily maximum temperature at least 5 °C higher than the average maximum temperature, have been recently foreseen to become particularly frequent and intense in Portugal. Therefore, agriculture constitutes one of the most sensitive sectors that could be affected by climate change. Among the xenobiotics contaminating agricultural crops, mycotoxins are the most challenging, since their presence represents an economic burden due to crops’ loss and serious health effects related to humans and animals [59].

## Therapeutic Challenges and Advances

Portugal relies on a limited arsenal of antifungal agents, including azoles, echinocandins, and amphotericin B. While azoles remain the first-line treatment for aspergillosis and candidemia, their efficacy is increasingly compromised by resistance. Echinocandins are effective against most *Candida* species but have limited activity against *Aspergillus*. Amphotericin B, despite its broad-spectrum activity, is associated with significant (nephro)toxicity, restricting its use to severe or refractory cases. Emerging antifungal therapies, such as olorofim and ibrexafungerp, offer hope for addressing resistance and expanding treatment options. Clinical trials are evaluating the efficacy of these novel agents, while combination therapies targeting multiple pathways are being explored to enhance treatment outcomes. Personalized medicine approaches, including genotypic profiling of pathogens and monitoring host immune responses, are also gaining attention as strategies to optimize antifungal therapy. In this context, Portuguese researchers are actively participating in immunotherapy trials for candidemia and influenza-associated pulmonary aspergillosis (IAPA). These trials aim to personalize immunotherapy by integrating selected clinical, immunological, genetic, and metabolic features of the patients. Although final data is not yet available, these studies represent the first worldwide efforts to focus on personalized, host-directed medicine approaches for invasive fungal diseases. Moreover, researchers in Portugal are leading various international multicenter initiatives to identify and functionally characterize individual host-derived factors that predict the risk and clinical progression of invasive mold infections in hematological patients, and that may also serve as innovative therapeutic targets for immunomodulatory strategies. In this regard, a recent trial involving Portuguese researchers has been launched to evaluate the clinical applicability of individual genetic risk factors in guiding antifungal prophylaxis for critically ill patients, aiming to prevent the onset of IAPA.

Identification of species included in section *Fumigati* remains challenging. The golden-standard approach – culturing methods – is not sufficient for an accurate identification of these species. Moreover, MALDI-TOF databases are still incomplete regarding the specific identification of most cryptic species (excluding *A. lentulus*), which leads to sequencing methods of specific genes to be the go-to option regarding this identification. Until this date, there is no available kit to identify these species through qPCR approaches. This methodology is considered the most appropriate regarding cost-effectiveness and sample handling (few hands-on time after DNA extraction), leading to less possible contaminations. Another important aspect to consider is that, due to COVID-19 pandemic, most health facilities have acquired a qPCR equipment, which make this type of identification more appealing [60, 61]. Therefore, on-going work aims the development of a qPCR assay to specifically identify, and distinguish, the most clinically relevant species in section *Fumigati* (*A. fumigatus* sensu stricto, *A. lentulus*, *A. udagawae* and *A. felis*). The key idea is to design specific primers, for each species, using genes that are known to distinguish these species by sequencing, employing melting curve analysis to specifically identify the isolates. Preliminary results showed that species included were able to be distinguished by melting temperature values, with no cross amplifications. Further steps include the optimization of the method to a multiplex reaction that will be amenable, not only to be used in clinical samples, but also to be used in fungal cultures, to facilitate the identification in Surveillance Programs. The development of this method will significantly reduce the turn-around time related to these species’ identification, and the cost associated, when comparing it to the present go-to method, i.e. sequencing.

To gain information on the current state of research on *C. albicans* in Portugal supporting a One Health approach in the context of candidiasis originating from this species, a non-exhaustive bibliographical search was carried out on works published mainly by Portuguese researchers, in the last 24 years and with open-access, and whose correspondent authors were affiliated with Portuguese institutions. This effort created a database with more than 70 articles, covering the main groups working in this area. In the north, groups in Braga and the Oporto area focus on the molecular and genetic basis of virulence, biofilm formation, and resistance mechanisms, as well as the discovery of antifungal compounds from natural sources. In Vila-Real and Bragança, efforts are directed toward plant-based and natural compound therapies to inhibit *Candida* infections. Regions such as Aveiro and Coimbra emphasize genetic regulation, host–pathogen interactions, and bioinformatics approaches to uncover virulence determinants and treatment targets, often integrating novel drug delivery strategies. Teams at Beira-Interior and in Leiria investigate clinical aspects, especially recurrent infections and gynecological candidiasis, with attention to resistance and natural antifungal agents. In the Lisbon area, research spans clinical epidemiology and antifungal resistance, molecular pathogenesis, antifungal drug development, host interaction studies, with a strong emphasis on medicinal chemistry and innovative therapeutic strategies. Together, these efforts reflect a robust and diverse national research landscape dedicated to understanding and combating candidiasis. Recent studies began to create the basis for a more integrated approach that aims to inform clinicians about the characteristics of each *C. albicans* clinical isolate, by detecting its relevant genetic determinants and hence allowing more personalized approaches for candidiasis. The identification of genomic factors for resistance to antifungals and overall virulence, is based in the literature but is also being searched de novo, using methodologies of population genomics to explore a large number of *C. albicans* isolates or strains of nationwide origin.

To this end, collections from different geographic origins are being characterized according to their resistance to antifungals, but also to the ability to produce biofilms or to respond to macrophages. At the same time, the genomes from the same strains are being sequenced to map the most prevalent causal genomic variants in the country and associate them with the respective phenotypes. It is hoped that this initiative, together with that of other Portuguese groups that use their own expertise to study *C. albicans*, can constitute the starting point for the creation of an effective program for candidemia surveillance in Portugal. Specifically, we emphasize the integration of nationwide strain collections and associated phenotypic/genotypic datasets, which can be consolidated into a centralized surveillance platform. Also, the use of standardized phenotyping protocols (e.g., biofilm formation, macrophage assays, antifungal susceptibility testing) across multiple research groups, and increasing local access to whole-genome sequencing platforms and bioinformatics tools will support harmonized data collection that is essential for large-scale pathogen monitoring. Other key step for an effective candidemia surveillance program in Portugal include the active participation in international consortia and One Health initiatives, which ensure alignment with global standards and access to collaborative expertise. This coordinated infrastructure represents a critical step toward evidence-based public health strategies to reduce the burden of invasive candidiasis.

## Other Fungal Challenges

Portugal faces significant challenges from pathogenic or opportunist species contributing to acquired fungal infections.

Approximately 90% of *Pneumocystis* spp. severe pneumonia (PCP or pneumocystosis) occur in people living with HIV (PLHIV) with CD4^+^ T cells counts < 200/mm^3^, and the incidence in Western Europe and the United States is < 1 case per 100 person-year [62] In Portugal, in the year 2022, in 138 cases of AIDS, PCP was the first opportunistic infection, diagnosed in 26.8% of the cases [63]. Currently, despite the success of PCP chemoprophylaxis and antiretroviral therapy (ART), this disease continues to cause morbidity and mortality among PLHIV. Most of these recent cases occur in patients who are unaware of their HIV-status, those with active substance abuse or psychiatric illness, those who are not receiving or responding to ART or prophylaxis, and in those with advanced immunodeficiency (CD4^+^ T < 100 cells/mm^3^) [64]. Furthermore, in recent years we have seen an increase in the number of PCP cases in non-HIV immunocompromised patients. Real-time quantitative PCR is the only technique suitable for a quantitative diagnosis, and these results have been used to differentiate PCP (active disease – medium–high fungal load) from carriage/colonization (low fungal load) [64, 65]. For a less invasive diagnosis of PCP, there are other laboratory strategies, which are based on the measurement of blood biomarkers that reflect the host–pathogen interaction, such as (1–3)-β-D-Glucan (the most used), Krebs von den Lungen-6 antigen, lactate dehydrogenase and S-adenosylmethionine [64, 65]. More recently, approaches based on the detection of specific anti-*P. jirovecii* antibodies in patients’ serum have shown encouraging results, allowing faster and cheaper screening and diagnosis of PCP. This in turn is helping to improve disease control and providing greater containment to healthcare systems [64, 66].

Also, considering the emergence of *Fusarium* as etiological agent of invasive mycosis and its major relevance due to it antifungal resistance profile and its role considering the One Health perspective [67], this genus should also be one of our major focus in further studies.

Other fungal infection that represents a challenge is histoplasmosis, an emerging concern in Portugal due to increased migration and travel from endemic areas. Portugal’s historical and demographic context increases its vulnerability to histoplasmosis. The country has strong migratory ties with endemic regions such as Brazil and Portuguese-speaking African countries [7, 68]. Additionally, the mass return of Portuguese citizens from African colonies in the 1970s and ongoing immigration from endemic areas elevate the likelihood of imported cases. Histoplasmosis remains underdiagnosed in Portugal due to overlapping symptoms with tuberculosis and limited diagnostic resources. As migration patterns persist and intercontinental travel grows, Portugal faces an increased risk of histoplasmosis cases. Once more, enhanced surveillance, awareness, and diagnostic tools are crucial to address this emerging public health concern and ensure timely management.

## Conclusions and Recommendations

A robust strategic framework must begin with coordinated surveillance efforts, encompassing sampling campaigns in workplaces and critical indoor environments such as healthcare settings. These efforts should be supported by enhanced laboratory capacity and diagnostic capabilities, enabling timely detection and response to fungal pathogens and antifungal resistance (fAMR). Despite the significant burden of deaths caused by fungal infections, clinical mycology remains critically underfunded. To strengthen national preparedness, the Portuguese Association of Medical Mycology (ASPOMM) strongly advocates that government and funding organizations should allocate more resources to the national response to fungal infections and antifungal resistance, focusing on fungal research, healthcare infrastructure, and public health initiatives. This includes aligning with WHO FPPL recommendations and establishing a legal framework that supports broad antimicrobial resistance (AMR) interventions, with a specific focus on fAMR.

Public engagement is also essential. Raising awareness and involving citizens in science can foster a culture of prevention and shared responsibility. Educational campaigns and community-based initiatives can enhance understanding and promote behavioral changes that reduce exposure and transmission. For example, regulating agricultural practices to limit azole fungicide use can also reduce environmental selection pressures and curb the spread of resistance, along with reducing outfall of non-quaternarily treated wastewater as most antifungals are not removed in primary to tertiary treatments and end up in the environment also. Additionally, occupational exposure to fungal pathogens is also an issue of concern and a significant risk for farmers, waste management industry workers, and construction workers, who are frequently exposed to contaminated materials.

Innovation must be a cornerstone of this strategy. Advancing translational and clinical research to identify novel fungal and host-derived biomarkers will complement existing diagnostic and therapeutic tools. Stimulating research and development (R&D) in fungal diagnostics, therapeutics, and environmental management is vital for long-term resilience.

To operationalize this holistic vision, we propose reinforcing the activities of ASPOMM, bringing together stakeholders from clinical, laboratory, veterinary, and environmental health sectors. This collaborative structure would focus on three interconnected domains:

Surveillance – Coordinated monitoring of exposure and fungal infections and resistance patterns.

Prevention – Environmental management, professional training, and public education.

Innovation – Support for R&D in diagnostics, therapeutics, and public health strategies.

By integrating these elements, we can build a comprehensive and sustainable response to fungal infections, ensuring better health outcomes, preparedness for future challenges and protect public health within a comprehensive One Health framework.

## Data Availability

No new data were created.
